# Notes and Notices

**Published:** 1889-07

**Authors:** 


FOR SALE.—A full set of eight volumes of THE HOMŒOPATHIC PHYSICIAN have been forwarded to us for sale. Price, $2.50 per volume. Single volumes will not be sold. Money must accompany the order. Postage extra to foreign countries. Address The Homœopathic Physician, 1123 Spruce Street, Philadelphia, Pa.

The American Public Health Association will holds its seventeenth annual meeting at Brooklyn, N. Y., October 22d, 23d, 24th, and 25th, 1889. The Executive Committee have selected the following topics for consideration at said meeting: I. The Causes and Prevention of Infant Mortality. II. Railway Sanitation. (a) Heating and ventilation of railway passenger coaches. (b) Water-supply, water-closets, etc. (c) Carrying passengers infected with communicable diseases. III. Steamship Sanitation. IV. Methods of Scientific Cooking. V. Yellow Fever. (a) The unprotected avenues through which yellow fever is liable to be brought into the United States. (b) The sanitary requirements necessary to render a town or city proof against an epidemic of yellow fever. (c) The course to be taken by local health authorities upon the outbreak of yellow fever. VI. The Prevention and Restriction of Tuberculosis in Man. VII. Methods of Prevention of Diphtheria, with Results of such Methods. VIII. How far should Health Authorities be permitted to apply known Preventive Measures for the control of Diphtheria. IX. Compulsory Vaccination. X. Sanitation of Asylums, Prisons, Jails, and other Eleemosynary Institutions. Papers upon miscellaneous sanitary subjects not included in the above list will be received by the Executive Committee, subject to the requirements of the By-Laws.

Department of the Interior, Census Office,

Washington, D. C., May 1st, 1889.

To the Medical Profession:—The various medical associations and the medical profession will be glad to learn that Dr. John S. Billings, Surgeon U. S. Army, has consented to take charge of the Report on the Mortality and Vital Statistics of the United States as returned by the Eleventh Census. As the United States has no system of registration of vital statistics, such as is relied upon by other civilized nations for the purpose of ascertaining the actual movement of population, our census affords the only opportunity of obtaining near an approximate estimate of the birth and death rates of much the larger part of the country, which is entirely unprovided with any satisfactory system of State and municipal registration. In view of this, the Census Office, during the month of May this year, will issue to the medical profession throughout the country "Physicians' Registers" for the purpose of obtaining more accurate returns of deaths than it is possible for the enumerators to make. It is earnestly hoped that physicians in every part of the country will co-operate with the Census Office in this important work. The record should be kept from June 1st, 1889, to May 31st, 1890. Nearly 26,000 of these registration books were filled up and returned to the office in 1880, and nearly all of them used for statistical purposes. It is hoped that double this number will be obtained for the Eleventh Census. Physicians not receiving Registers can obtain them by sending their names and addresses to the Census Office, and, with the Register, an official envelope which requires no stamp will be provided for their return to Washington. If all medical and surgical practitioners throughout the country will lend their aid, the mortality and vital statistics of the Eleventh Census will be more comprehensive and complete than they have ever been. Every physician should take a personal pride in having this report as full and accurate as it is possible to make it. It is hereby promised that all information obtained through this source shall be held strictly confidential.

Robert P. Porter, *Superintendent of Census.*

